# Patterns of diarrhoeal disease among under-five children in Plateau State, Nigeria, 2013–2017

**DOI:** 10.1186/s12889-021-12110-y

**Published:** 2021-11-13

**Authors:** Joseph Chikan Jiwok, Ayo Stephen Adebowale, Idongesit Wilson, Vijaya Kancherla, Chukwuma David Umeokonkwo

**Affiliations:** 1 Nigeria Field Epidemiology and Laboratory Training Programme, Abuja, Nigeria; 2 Department of Community Medicine, Ahmadu Bello University, Zaria, Nigeria; 3 Department of Epidemiology and Medical Statistics, University of Ibadan, Ibadan, Nigeria; 4 African Field Epidemiology Network, Abuja, Nigeria; 5 Department of Epidemiology, Emory University Rollins School of Public Health, Atlanta, GA USA; 6 Department of Community Medicine, Alex Ekwueme Federal University Teaching Hospital, Abakaliki, Ebonyi State Nigeria

**Keywords:** Diarrhoea, Trends, Under-five children, Plateau state, Nigeria

## Abstract

**Background:**

Diarrhoea is the second commonest cause of under-five mortality accounting for over half a million deaths annually. Although the prevalence of diarrhoea in Plateau State is lower than the national figure, the level remains high despite remarkable progress in the reduction of under-five mortality. This study seeks to determine the pattern of diarrhoea disease among under-fives in Plateau State.

**Methods:**

We extracted data from the Integrated Disease Surveillance and Response platform between January 2013 and December 2017 and analysed the trends of diarrhoea, age-specific case fatality rate (ASCFR), and seasonal patterns. We modelled the quarterly pattern of diarrhoea cases using additive time series and predicted the expected cases for 2018–2020.

**Results:**

We documented 60,935 cases of diarrhoea with age group 12–59 months having the highest number of cases (49.3%). The age group < 1 month had the highest ASCFR of 0.53%. Seasonal variation showed cases peaked in the first and third quarters of each year, except for the year 2016. The time series projection estimated 16,256, 17,645 and 19,034 cases in the year 2018, 2019 and 2020 respectively.

**Conclusion:**

Seasonal variation exists, and trends show an increased pattern of diarrhoeal disease among under-fives. There is a need to strengthen the implementation of diarrhoeal preventive and control strategy in the state and to improve the quality of data reporting.

## Background

Diarrhoea is the second commonest cause of childhood mortality among children under 5 years of age [1]. In developing countries, children under 3 years of age experience on average three episodes of diarrhoea every year. And each episode contributes to a significant nutritional deprivation which negatively affects child growth [2].

Diarrhoea disease is the leading cause of infant and child mortality in developing countries, and about 1.8 million children die per annum from this disease. These numbers of diarrhoeal deaths are still high despite a fall in childhood diarrhoeal disease from 4.6 million to 0.8 million over the last three decades [3–5]. Young children are especially vulnerable to diarrhoeal disease, and most deaths related to diarrhoea took place in Africa and South Asia [6]. The World Health Organization (WHO) estimates that 525,000 under-five children die globally because of diarrhoeal diseases each year, with 1.7 billion cases of diarrhoeal disease diagnosed annually [1].

The mortality of diarrhoea remains high in Africa despite being easily treated with oral rehydration therapy [7]. According to WHO, in Southeast Asia and Africa, diarrhoea accounts for as much as 8.5 and 7.7% of all deaths respectively [8].There has been a decline in mortality rates from diarrhoea in the past three decades, however there is no improvement in sub-Saharan Africa and South Asia where 90% of diarrhoeal deaths occured [9].

In Africa, they estimated every child has five episodes of diarrhoea per year and that 800,000 children die each year from diarrhoea and dehydration. Diarrhoea is the second biggest killer of children in Nigeria, responsible for about 16% of child’s death every year. An estimated 151,700 children die in Nigeria every year from diarrhoea disease [8]. A high death rate because of diarrhoea can undermine the accomplishment of the Sustainable Development Goals (SDGs) in Plateau State and Nigeria if left unchecked.

To reduce diarrhoea mortality and morbidity, studies have helped inform intervention programs especially before the rainy season in regions with majority of cases [10, 11], allocation of limited resources and securing commodity supply chain to avoid stock outs [5], training and deployment of staff to remote and hard to reach communities [12].

In Nigeria, there is regional variation in the distribution of diarrhoea. A national study conducted in Nigeria showed a higher prevalence of the diarrhoeal disease among infants in the northern (37.7%) part of Nigeria of which Plateau State is located compared to the southern part (21.1%) [6]. The diarrhoea prevalence rate among under-five children in Nigeria is 10% [13]. There are a lot of vulnerable children at risk of death from diarrhoea in Nigeria [3]. The increasing disease burden and limited research in Plateau State suggests the need for our study in order to help inform health policies that will promote the control of diarrhoeal disease among under-fives.

Diarrhoea disease is associated with high under-five mortality rate (U5MR) in Nigeria, a country with U5MR of 128 per 1000 [13]. We conducted this study in Plateau State which is known to have high risk of diarrhoeal disease in Nigeria [13]. Studies have shown an association between poor housing, crowded conditions, low income and a higher rate of diarrhoea [14]. Others include poor storage of drinking water, use of unsafe water sources, improper disposal of children’s faeces and household garbage. Also implicated are poor hand hygiene practices among nursing mothers, partially breastfed children, bottle-feeding, malnutrition and immunodeficiency [14].

The aims of this study are to determine the; trend, case fatality rate, seasonal pattern and employ a time series model to predict expected cases of diarrhoeal disease among under-five children in Plateau State for 2018–2020. Time series forecasting is an important aspect of descriptive analysis and presentation of disease like diarrhoea, which is often neglected in Nigeria. This analytical approach is crucial because predicting diarrhoeal disease involves a time component because of the seasonal nature of the disease. The expected quarterly projection of children under the age of 5 years who will have diarrhoea in the study area in years ahead is necessary for monitoring and assessment of disease situation to inform policy. It will also enhance the level of preparedness towards plans to mitigate the spread of the disease.

## Methods

### Study design

We conducted a retrospective secondary data analysis of IDSR diarrhoeal disease data among under-five children in Plateau State. We extracted data from the Integrated Disease Surveillance and Response (IDSR) platform, form 003 from January 2013 to December 2017. The IDSR 003 tool collects monthly data on forty (40) priority health events including diarrhoea. They collect this data from health facilities and sent to Local Government Area (LGA) Disease Surveillance and Notification Officer (DSNO) who collates the data and transmits to the State DSNO who transmits this to the Federal Ministry of Health (FMOH) monthly. Feedback is through the same channels.

### Study setting

Plateau State is in the North-central zone of Nigeria with an estimated population of four million people, of which over seven hundred thousand (17.5%) are children under 5 years of age. It has 17 LGAs and the predominant occupation of its people is agriculture. It has a near temperate climate with an average temperature of between 13 and 22 degree centigrade while the annual rainfall varies from 131.75 cm in the southern part to 146 cm on the Plateau [15].

There are about 1000 health facilities in the State which comprises both government and private-owned facilities. These include 3 tertiary, 59 secondary and 940 primary health care facilities. Its health facility per population is 1.4/1000 (2/1000- WHO benchmark). Source of water in rural communities is mainly streams, rivers and hand-dug wells.

### Description of IDSR and information flow

The IDSR incorporates the surveillance of diseases. It has an organized data stream and a feedback mechanism at various levels of the health system framework (community, health facilities, LGA, state to national levels).

Patients from the community come to the health facility that offers care, and they enter patient’s data in a register. The data is shared with the LGA, state and national periodically. Some diseases are reported weekly, monthly and quarterly, but those of high epidemic potential or targeted for elimination are reported immediately.

At the LGA level, data are collected from the various health facilities monthly and shared with the state, that aggregates these data from the various LGA and communicate same with the national level (Federal Epidemiology Division). The national gives feedback to the lower levels on data analysis, surveillance performance indicators and laboratory results [16].

### Timeline for reporting

Suspected cases for immediate reportable diseases ought to reach the LGA within 48 h seen at the health facility. Health facilities send weekly reports to the LGA on the first working day (Monday) of the following week. The LGAs are to collate same and forward to the State by the second working day (Tuesday) of the following week. The State forwarded weekly data to the Federal Epidemiology Division by the third working day (Wednesday) after the reporting week [16].

Monthly reports from the health facility should get to the LGA by the first week after the reporting month. The LGA compiles data and forward same to the State by the end of the second week of the succeeding month. The State should compile data from various LGAs and forwarded to the Federal Epidemiology Division by the third week of the succeeding month.

Duplicates of these reports are shared with the Health Management Information System (HMIS) unit of the State Ministry of Health (SMOH) and of the FMOH department of health planning research and statistics by the State and Federal Epidemiology Division, respectively [16].

### Check data quality

At each level or reporting site, the surveillance team acknowledges receipt of the report, sign in to an appropriate log book of any information or surveillance report received from any reporting site, audit the information quality, confirm whether the form (printed version or electronic record) is precisely and totally archived, check to make certain there are no inconsistencies on the form, record in the log the date the information showed up, type of information and who sent it. Check whether the information index showed up timely or late. Merge the information and store them in a database. They receive written reports, review case-based reporting form to see if any fundamental data is absent. If reports are not received or are consistently late, contact or visit the health facility to know the cause. Liaise with the staff at the reporting site to assist with solution that could improve reporting [16].

### Data source and management

We received diarrhoea surveillance data from the Plateau State Integrated Disease and Surveillance Response (IDSR) platform. The source of the data had 100% completeness for the variables of interest. We extracted data on age, cases, disease outcome, the number of cases per month from the surveillance system. The IDSR aggregated the age in children under-5 years into three categories: < 1 month, 1–11 month(s) and 12–59 months.

Data was cleaned and analysed to describe the trend, case fatality rate, the seasonal pattern of diarrhoeal disease and predicted cases using Microsoft Excel and SPSS and presented as frequencies and graph at 95% confidence interval. We plotted the patterns of diarrhoea to establish seasonality in the reported number of cases from 2013 to 2017. A time series model was used to observe the pattern of variation.

Series (yt) can be represented by a moving average level that changes over time according to the following equation: *yt* = *TRt* + *SVt* + *it* [17]

where yt is the observed value of the diarrhoea cases in time period t;

TRt is the trend in time period t obtained by the moving average method;

SVt is the seasonal factor in time period t; and

*it* is the irregular factor. We assumed *it* satisfies the usual regression assumptions of constant variance, independence and normality.

A three-month period moving average was estimated for yt and the trend line was determined to average the irregular variation in the data. Thus, the estimate of TRt is:

$$ TRt= yt-\frac{\left(1+ yt+ yt+1\right)}{3} $$

using the above assumption,

$$ it=0\kern0.75em \mathrm{and}\  SVt= yt- TRt. $$

### Ethical consideration

This is a secondary data analysis of routinely collected data. Permission was sought from Plateau State Specialist Hospital research and ethics committee for the use of the data. The data did not include personal identifiers.

## Results

The IDSR reported 60,932 cases of diarrhoea in Plateau State between 2013 and 2017 (Table 1). The age group of 12–59 months had the highest reported cases, 30,053 (49.3%). Age-specific case fatality rate was highest (0.53%) in children < 1 month and lowest (0.16%) in children 12–59 months (Table 2).

**Table 1:** Annual reported cases of diarrhoea among under five in Plateau State, 2013–2017

Years	Cases	Percentage
2013	8027	13.0
2014	11,824	19.0
2015	12,783	21.0
2016	13,841	23.0
2017	14,460	24.0
**Total**	60,935

**Table 2:** Age distribution and age specific case fatality rate of diarrhoeal cases among under-five children in Plateau State, 2013–2017

Age group Month(s)	Frequency (***N*** = 60,935)	Percentage (%)	Death (***N*** = 132)	Case fatality rate (%)	95% C. I of CFR	
					Lower	Lower
< 1	5899	9.7	31	0.53	0.34	0.71
1–11	24,983	41.0	52	0.21	0.15	0.26
12–59	30,053	49.3	49	0.16	0.12	0.21

**Fig. 1:**
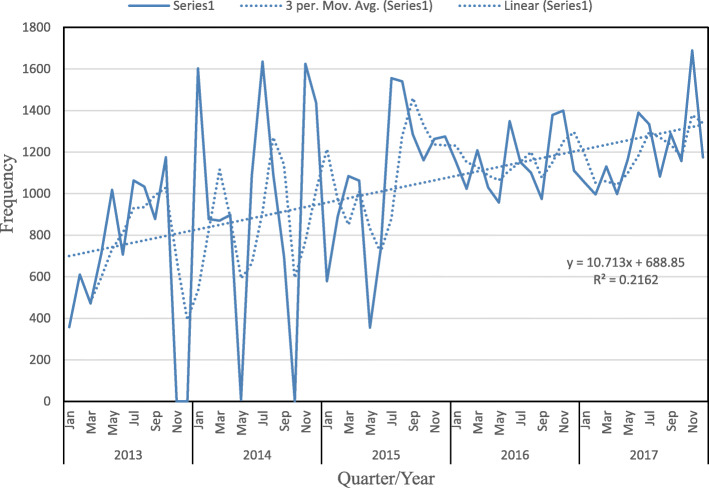
Frequency distribution of observed and 3-month moving average of cases of diarrhoea among under-fives in Plateau State, 2013–2017

There is high positive variation in the third quarterly period during each year, except in 2016, when only a negative variation of − 256.67 reported cases of diarrhoea occurred (Table 3). Frequency distribution of observed and 3 months moving average of cases of diarrhoea in Plateau State across the year showed an upward trend with a trend line slope of 0.02162 (Fig. 1). The pattern of this variation follows the time series additive model.

**Table 3:** Estimation of trend and seasonal variation of diarrhoea cases in Plateau State in each quarter of the study period

Year	Quarter	Number of diarrhoea cases (y_**L**_)	3-Qtr Moving Total	3-Qtr Moving average Trend (T_**L**_)	Seasonal variation SV= (y_**L**_-T_**L**_)
**2013**	Q1	1439			
	Q2	2437	6852	**2284.00**	153.00
	Q3	2976	6588	2196.00	780.00
	Q4	1175	7501	2500.33	− 1325.33
**2014**	Q1	3350	6515	2171.67	1178.33
	Q2	1990	8762	2920.67	− 930.67
	Q3	3422	8474	2824.67	597.33
	Q4	3062	9036	3012.00	50.00
**2015**	Q1	2552	7765	2588.33	−36.33
	Q2	2151	9084	3028.00	− 877.00
	Q3	4381	10,231	3410.33	970.67
	Q4	3699	11,467	3822.33	− 123.33
**2016**	Q1	3387	10,422	3474.00	−87.00
	Q2	3336	9951	3317.00	19.00
	Q3	3228	10,454	3484.67	−256.67
	Q4	3890	10,298	3432.67	457.33
**2017**	Q1	3180	10,624	3541.33	− 361.33
	Q2	3554	10,440	3480.00	74.00
	Q3	3706	11,280	**3760.00**	−54.00
	Q4	4020			

*ITLPQ* Increase in trend line per quarter

The seasonal variation of diarrhoea by a quarter in Plateau State between 2013 and 2017 was de-seasonalised and thereafter adjusted to find out what the exact variation should be for the quarterly periods (January–March, April–June, July–September and October–December) of any year. The data show that higher seasonal variation for quarterly periods 1 (Q1 = 222.1319) and 3 (Q3 = 500.2431), than quarterly periods 2 (Q2 = -399.507) and 4 (Q4 = -322.868). Increase in trend line per quarter is (3760–2284)/ (18-1) = 86.82353 (Table 4)

**Table 4:** De-seasonalisation of seasonal varaiation of diarrhoea by quarter in Plateau State 2013–2017, adjusted to ascertain what the exact variation should be for the quarterly periods (January–March, April–June, July–September and October–December) of any year

Year	Q1	Q2	Q3	Q4	
2013	–	153.00	780.00	−1325.33	
2014	1178.33	−930.67	597.33	50.00	
2015	−36.33	− 877.00	970.67	−123.33	
2016	−87.00	19.00	−256.67	457.33	
2017	−361.33	74.00	−54.00	–	
Total	693.6667	− 1561.67	2037.33	− 941.33	
Average	231.2222	− 390.417	509.3333	− 313.778	36.36111
					9.090278
	9.090278	9.090278	9.090278	9.090278	
Quarterly variation	222.1319	−399.507	500.2431	−322.868	
ITLPQ^a^	86.82353				

^a^*ITLPQ* Increase in trend line per quarter

**Fig. 2:**
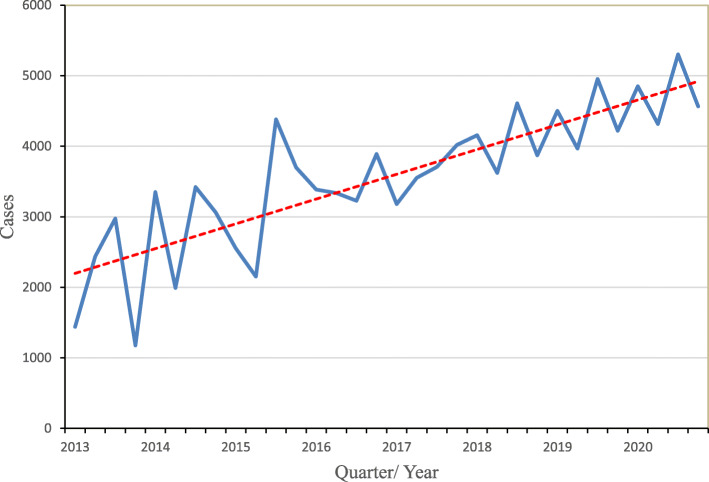
Available and projected data of diarrhoea among under five in Plateau State, 2013–2020

In a three-year projection of cases of diarrhoea among under-fives in Plateau State, the first and third quarter predicts a rise in cases compared to the second and fourth quarters (Table 5). Available and projected data of diarrhoeal disease in Plateau State 2013–2020 shows an upward trend (Fig. 2).

**Table 5:** Three-year projection of cases of diarrhoea among under-fives in Plateau State

Year	Quarters	TL	ITLPQ	Cumm	Quarterly variation	Projection	95% C. I for PV	
							Lower	Upper
**2017**	Q1							
	Q2							
	Q3	3760						
	Q4		86.82353	3846.824	−322.868	**3524**	3408	3640
**2018**	Q1		86.82353	3933.647	222.1319	**4156**	4030	4282
	Q2		86.82353	4020.471	−399.507	**3621**	3503	3739
	Q3		86.82353	4107.294	500.2431	**4608**	4475	4741
	Q4		86.82353	4194.118	−322.868	**3871**	3749	3993
**2019**	Q1		86.82353	4280.941	222.1319	**4503**	4371	4635
	Q2		86.82353	4367.765	−399.507	**3968**	3845	4091
	Q3		86.82353	4454.588	500.2431	**4955**	4817	5093
	Q4		86.82353	4541.412	−322.868	**4219**	4092	4346
**2020**	Q1		86.82353	4628.235	222.1319	**4850**	4714	4986
	Q2		86.82353	4715.059	−399.507	**4316**	4187	4445
	Q3		86.82353	4801.882	500.2431	**5302**	5159	5445
	Q4		86.82353	4888.706	−322.868	**4566**	4434	4698

*C.I* Confidence Interval, *PV* Projected values

$$ \mathbf{ITLPQ}=\frac{\boldsymbol{Most}\ \boldsymbol{recent}\ \boldsymbol{trend}\ \boldsymbol{less}-\boldsymbol{opening}\ \boldsymbol{trend}\ }{\boldsymbol{Number}\ \boldsymbol{of}\ \boldsymbol{trend}-\mathbf{1}} $$

The 3Qtr Moving average Trend (T_L_) = Most recent trend less- opening trend illustrated in Table 3 is calculated thus:

Add up data from 2013 Q1, Q2, and Q3 and divide by 3. List the answer next to Q2. Repeat the process excluding 2013 Q1, now add Q2, Q3 and Q4 of 2013 divide by 3 and list answer next to Q3 2013. Repeat for all the Quarters till 2017 Q4. Note that there was no moving average for 2013 Q1 and 2017 Q4 due to insufficient data to calculate them.

$$ \mathbf{ITLPQ}=\frac{3760-2284}{18-1}=\frac{1476}{17}=86.82353 $$

## Discussion

This study found that diarrhoeal disease among under-five children occurs throughout the year. The most affected age group was 12–59 months. This contrast with an earlier work that reported the highest burden among the 0–11 months age group [10]. Findings from the Nigeria demographic and health survey (NDHS) show diarrhoea was most common among children age 12–23 months and least common among those aged 48–59 months [18]. Kenya Demographic and Health Survey (KDHS) shows that the prevalence of diarrhoea is highest in children aged between 6 and 11 months, followed closely by children between the ages of 12 and 23 months [19]. The higher rate in the older age group might be because of malnutrition, improper hygiene and inadequate care as compared with the younger age group who receive more care and attention by their mothers.

The highest annual cases of diarrhoea in this study was 24% in 2017, and the lowest was 13% in 2013. We may attribute the increasing trend of diarrhoea in this study to better capturing. NDHS national prevalence of diarrhoea among under-five was 10% while Plateau State was 5.6% [18]. Higher prevalence has been reported. Ucheh et al. reported an incidence of 51.8% [20], and Houatthongkham et al. (74.8% in children 0–24 months) [21].

The age-specific case fatality rate is highest in children < 1 month and lowest in the older age group 12–59 months. These findings are consistent with earlier studies in which mortality was highest in children below 1 year [22]. The high mortality in the younger age group in this study could be due to low immunity, poor caregiver hygiene practices and malnutrition. In Nigeria, only 17% of children under 6 months had exclusive breastfeeding [18]. Most infants are on complementary feeds and have lost maternal antibodies. They are active, crawl around, pick up and ingest contaminated objects. Malnutrition, open faecal disposal, poor hygiene, inadequate immunization coverage, lack of access to safe drinking water, insecurity and natural disasters are some predictors of mortality in children under 5 years of age.

This study depicted seasonal variation of diarrheal disease in this age group with most cases reported in the first and third quarters of the year having peaked in the dry and rainy seasons. This corroborates a study in Ghana that reported bimodal seasonality of diarrhoea. The second peak occurred between December and February before the rainy season (May to August) [10]. In a similar study in Bangladesh, the first peak occurs prior to the high rainfall period and the second peak occurs towards the end of the high rainfall [23]. Diarrhoea cases in China showed a bimodal distribution: diarrhoea in children less than 5 years was more likely to peak in fall-winter seasons, while diarrhoea in persons greater than 5 years peaked in summer [24]. Another study reported seasonal distribution of infectious diarrhoea in Shanghai with peaks in winter and summer.

Winter peaks were mainly caused by norovirus and rotavirus, while summer peaks were caused by bacterial infections [24]. Our study has the first peak between January to March prior to the rainy season and the second peak between July to September towards the end of the rainy season. The rainy season is usually characterised by heavy rainfall leading to flood, poor sanitation and contamination of water bodies from open or broken sewer. In Botswana, a study on climate change suggests that a drier, hotter climate might exhibit a positive influence on the dry seasonal diarrhoeal case incidence [4].

Based on our time series modelling, there was an increasing trend in the cases of diarrhoea among children under the age of 5 years during 2013–2017, and our projection showed that this trend will continue up until year 2020, all things being equal. Time-series data are useful for accurately monitoring of disease pattern over a period, and therefore, forecast trends. The assumption that the quality of current intervention to mitigate the disease would remain the same between 2018 and 2020 may not be true. This is because government and some international agencies put efforts in place to meet the goal of a reduction in the cases of diarrhoea in Nigeria. This increasing trend of diarrhoeal disease in Plateau State found in this study may be because of the likelihood that diarrhoea cases reporting mechanisms might just be getting better as clinics are improving and learning more about reporting strategies. The three-year projection of cases of diarrhoea among under-fives in Plateau State from 2018 to 2020 shows a rise in cases in an undulating pattern. The first and third quarter predict peaks in cases with a slight decline in the second and fourth quarters.

### Strengths and limitations

To our knowledge, this is the first study on the pattern of diarrhoeal disease among under-five children in Plateau State using a comprehensive national dataset (the IDSR platform data).

Despite the limitations, this study provides the policy maker value information to make better plans and projection.

The case-based surveillance system does not capture the cases who sought treatment outside formal health care or deaths which occur outside the health facilities. This may cause under reporting and so findings from this study cannot generalise the entire community.

Diarrhoeal disease is often reported in episodes and an individual might have over one episode within the same calendar period, however, these are consistent across the period and cautiously interpreted as it is a limitation in making analysis.

There might be misclassification bias of diarrhoea cases when healthcare providers make the wrong diagnosis as laboratory confirmation of specific agents causing diarrhoea is not routinely recommended for surveillance purposes.

## Conclusion

The most affected age group in this study are children 12–59 months and highest mortality in children < 1 month. Diarrhoea depicts bimodal seasonality with the highest peak in the rainy season. There is a need to implement prevention and control strategy at the State and improve data reporting. Additional studies at community level would be useful in reporting the number of episodes of diarrhoea and identify LGAs differences for intervention.

## Data Availability

The data that supports the findings of this study are available from Plateau State Ministry of Health, but restrictions apply to the availability of these data which were used under license for the current study and currently not publicly available. Data are however available from the authors upon reasonable request and with the permission of the Plateau State Ethical Committee.

## Abbreviations

ASCFR: Age specific case fatality rate
DSNO: Disease surveillance and notification officer
ELISA: Enzyme-linked immunosorbent assay
FMOH: Federal Ministry of Health
HMIS: Health Management Information System
IDSR: Integrated Disease Surveillance System
JUTH: Jos University Teaching Hospital
KDHS: Kenya Demographic and Health Survey
LGAs: Local Government Areas
NDHS: National Demographic and Health Survey
RT-PCR: Real time- polymerase chain reaction
SDGs: Sustainable Development Goals
SMOH: State Ministry of Health
WHO: World Health Organization
